# Hand Gesture Recognition Using EMG-IMU Signals and Deep Q-Networks

**DOI:** 10.3390/s22249613

**Published:** 2022-12-08

**Authors:** Juan Pablo Vásconez, Lorena Isabel Barona López, Ángel Leonardo Valdivieso Caraguay, Marco E. Benalcázar

**Affiliations:** Artificial Intelligence and Computer Vision Research Lab, Escuela Politécnica Nacional, Quito 170517, Ecuador

**Keywords:** hand gesture recognition, electromyography, inertial measurement unit, reinforcement learning, deep Q-network

## Abstract

Hand gesture recognition systems (HGR) based on electromyography signals (EMGs) and inertial measurement unit signals (IMUs) have been studied for different applications in recent years. Most commonly, cutting-edge HGR methods are based on supervised machine learning methods. However, the potential benefits of reinforcement learning (RL) techniques have shown that these techniques could be a viable option for classifying EMGs. Methods based on RL have several advantages such as promising classification performance and online learning from experience. In this work, we developed an HGR system made up of the following stages: pre-processing, feature extraction, classification, and post-processing. For the classification stage, we built an RL-based agent capable of learning to classify and recognize eleven hand gestures—five static and six dynamic—using a deep Q-network (DQN) algorithm based on EMG and IMU information. The proposed system uses a feed-forward artificial neural network (ANN) for the representation of the agent policy. We carried out the same experiments with two different types of sensors to compare their performance, which are the Myo armband sensor and the G-force sensor. We performed experiments using training, validation, and test set distributions, and the results were evaluated for user-specific HGR models. The final accuracy results demonstrated that the best model was able to reach up to 97.50%±1.13% and 88.15%±2.84% for the classification and recognition, respectively, with regard to static gestures, and 98.95%±0.62% and 90.47%±4.57% for the classification and recognition, respectively, with regard to dynamic gestures with the Myo armband sensor. The results obtained in this work demonstrated that RL methods such as the DQN are capable of learning a policy from online experience to classify and recognize static and dynamic gestures using EMG and IMU signals.

## 1. Introduction

In recent years, the use of non-verbal communication techniques has proven useful for creating human–machine interfaces (HMIs). In particular, hand gesture recognition (HGR) systems have been used in applications such as sign language recognition, human–machine interfaces, muscle rehabilitation systems, prosthesis design, robotic applications, and augmented reality, among others [1,2,3,4,5,6]. However, designing HGR systems that are capable of determining with high accuracy the moment a certain gesture was performed is a challenging problem. This is due in part to the variability of the signals of each gesture between different users, as well as the similarities that the signals of different hand gestures may have.

Several HGR systems use vision-based methods, for example, Kinect [7] and Leap Motion Sensor [8]. On the other hand, sensor-based HGR systems typically use gloves with inertial measurement units (IMU) [9,10], as well as non-invasive surface electromyography (EMG) methods for the detection of arm muscle activity, such as the G-force and Myo armband sensors [6]. However, the performance of vision-based method systems can be affected by occlusion and illumination issues, as well as the distance between the sensor and the hand. For this, sensor-based HGR systems based on EMG or IMU signals are preferred for different HGR applications. It is worth mentioning that EMG signals (EMG) are often selected when static gestures are used since the information from muscle activity is usually sufficient to characterize this type of hand gesture [1,4,5]. On the other hand, IMU signals (IMUs) are usually selected to characterize dynamic gestures since this type of gesture primarily depends on hand and arm movements [6]. Therefore, a combination of EMG and IMU signals to recognize static and dynamic hand gestures could increase the performance of HGR systems since more information is analyzed for each gesture [11]. However, this is still an open research problem [12,13].

EMG signals can be modeled as a stochastic process that depends on whether the muscle contraction is static or dynamic. However, to address these problems, machine learning (ML) and deep learning (DL) techniques have been commonly used to classify and recognize EMG signals instead of mathematical models since the latter have high design complexity and performance issues [1,14]. In particular, supervised methods, such as support vector machines (SVMs), k-nearest neighbors (K-NNs), artificial neural networks (ANNs), convolutional neural networks (CNNs), a fusion of the transformer model and the CNN model (transformer-CNN), and long short-term memory (LSTM) networks, have shown high-performance results for HGR systems (at least 80% classification accuracy and 300 ms processing time) [1,15,16,17,18,19,20]. However, these models still require a fully labeled dataset to be trained, which makes them unsuitable for learning using new experiences gained online when the user interacts with the system. On the contrary, reinforcement learning (RL) approaches can help build models that learn online from experience. These models could help improve the performance of the HGR system over time since the system can adapt to each user in an online manner after each interaction with the system, which helps reduce the problem of interpersonal variability. Reinforcement learning methods are based on the maximization of the accumulated reward that is obtained by trying to correctly predict a gesture from online experiences, which allows for finding an optimal policy for an agent to use to predict categories of signals in a given environment [16].

There have been a few attempts to use RL techniques for HGR and arm movement or hand gesture characterization using sensor-based systems. For example, in [21], the authors used the Myo armband sensor to extract 9-axis IMU and 8-axis EMG sensor information to classify dynamic hand gestures using a deep Q-network (DQN) model. The experiment consisted only of three different hand gestures based on drawing a circle, a rectangle, and a triangle in the air. Each of these three gestures had 30 training data and 20 test data. The agent was built using a CNN with and without LSTM layers and was demonstrated to obtain high classification performance. In [22], the authors used the UCI dataset, which contains EMG data from six users performing six different hand gestures. From this dataset, time-domain features were obtained using a CNN-based automatic feature extraction method. To learn a classification policy, a deep Q-learning dueling technique was used, which allows for the selection of the most relevant characteristics throughout the training. The base dataset was composed of a total of 2700 EMG signal samples for the six hand gestures. As this was a sparse dataset, the authors used data augmentation methods using Gaussian noise, random horizontal flipping, and vertical flipping on the EMG data to obtain 10,000 samples. The authors showed that CNN performed better than ANN for this dataset. In another work, the authors proposed a classifier based on the neural reinforcement learning (NRL) method to classify finger movements using only EMGs [23]. For this, the authors used four feature extraction methods, which were the variance, mean absolute value, zero crossing, and waveform length of seven different gesture classes. Then, they used a k-nearest neighbor classifier based on reinforcement learning to classify the extracted features using a trial-and-error approach. The authors performed experiments on 10 users with general and specific models, demonstrating that it was feasible for the NRL user to identify typing movements using EMG signals from the forearm. In [24], a reinforcement learning-based classifier capable of learning to classify arm and finger movements was designed. For this, a 26T System was used to obtain EMG signals from 10 subjects using 1, 2, and 3 electrodes, respectively, to compare their results. The temporal characteristics that were used were the length of the waveform, the mean absolute value, the variance, and the zero crossing. An algorithm based on Q-learning was used for the classification stage, where the agent was made up of an ANN to infer six classes of arm positions and four classes of finger movements. The authors used 144 training samples and 95 test samples to build specific models for each of the 10 subjects. Finally, we presented an approach to classify and recognize five different static hand gestures based only on the EMGs in [16]. For this, we used Q-learning with an ANN as a policy representation of the agent. However, we used only the EMG signals to recognize static gestures and data were obtained using only the Myo armband sensor. Although the results obtained were encouraging, it is still necessary to explore other types of gestures and sensor behaviors when using different RL-based methods. Moreover, the use of IMU is still key to recognizing dynamic gestures, and the combination of EMG-IMU signals still needs to be analyzed and compared to a case when only EMGs are used to develop HGR systems based on RL methods. In summary, the use of datasets with a considerable number of samples and participants for both dynamic and static gestures based on EMG and IMU information still needs to be explored for different RL-based methods and sensors. To the best of our knowledge, this work is the first attempt to use EMG-IMU signals from a large dataset from two different sensors (Myo armband and G-force) and compare the results with other methods.

Considering the literature review presented above, the main contributions of the present work are listed below:

- We use our large dataset composed of 85 users with information on 11 different hand gestures (5 static and 6 dynamic gestures) that contain EMG and IMU signals. The data were taken from two different armband sensors, the Myo armband and G-force sensors.
- We successfully combine the EMG-IMU signals with the deep Q-network (DQN) reinforcement learning algorithm. We propose an agent’s policy representations based on artificial neural networks (ANN).
- We compare the results of the proposed method using both sensors, the Myo armband and G-force sensors. We also compare the results found in the present work, which uses EMG and IMU signals, with those of a method previously developed on a dataset that used only EMG signals and the Q-learning algorithm.

The rest of this work is organized as follows. In Section 2, the proposed method for an HGR system based on EMG-IMU signals and RL is presented and each stage is explained in detail. The classification and recognition results of the proposed method are presented in Section 3. The discussion section is in Section 4. Finally, the conclusions are provided in Section 5.

## 2. Hand Gesture Recognition Method

**Figure 1:**
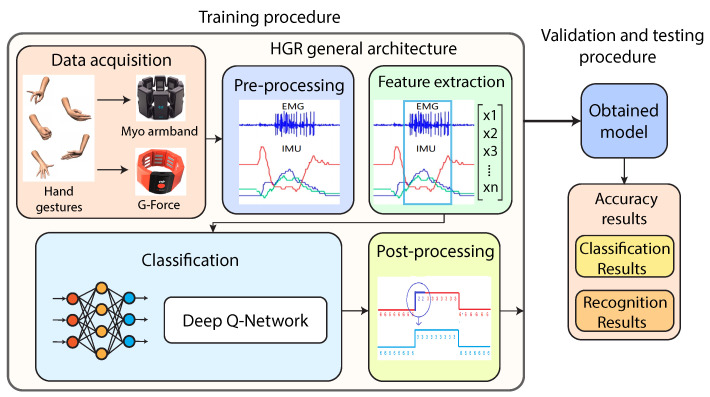
Hand gesture recognition method based on EMG-IMU and RL.

In this section, we present the proposed method for the HGR system based on EMG-IMU signals and RL (Figure 1). As can be observed, the proposed method is composed of data acquisition, pre-processing, feature extraction, classification (DQN), and postprocessing stages. The data were taken from two different armband sensors to compare results, which are the Myo armband and G-force sensors. We combined the EMG-IMU signals with the deep Q-network (DQN) reinforcement learning algorithm to develop the proposed HGR system. Next, we explain in detail each stage.

### 2.1. Data Acquisition

In this work, we use EMG-IMU data of 12 different hand gesture categories—11 different hand gestures and 1 relax gesture—in which 5 of them are static gestures—wave in, wave out, fist, open, and pinch—and the other 6 are dynamic gestures—up, down, left, right, forward, and backward. The data were collected using the Myo armband—a sensor with 8 channels at a sampling rate of 200 Hz—and the G-force armband—a sensor with 8 channels at a sampling rate of 1 kHz. The proposed dataset consists of 85 users, of whom 43 are used for training and validation to find the best possible hyperparameter configurations. From this group, 16 users are from the Myo armband sensor data and 27 from the G-force sensor data. On the other hand, 42 users are used for testing to evaluate overfitting and to calculate the final results. From this group, 16 users are from the Myo armband sensor data and 26 from the G-force sensor data. The data of each user in the training set is composed of 180 hand gesture repetitions—15 repetitions for each gesture—and the other 180 samples are for validation. This division of samples is similar to the test set. We summarize the dataset distribution for both the training and testing sets in Table 1. The dataset has been made public and is available at the following link https://laboratorio-ia.epn.edu.ec/en/resources/dataset/emg-imu-epn-100 accessed on 18 November 2022.

### 2.2. Pre-Processing

The preprocessing of each EMG sample consisted of using a sliding window on each sample to analyze it separately [1,14]. In this work, we chose a window length of 300 and a step of 40, where these values were selected based on experimentation to achieve high classification and recognition accuracy. Since we had two different sensors—Myo armband and G-force—with different sample frequencies—200 Hz and 1 kHz—a resampling was performed by applying an FIR antialiasing low-pass filter to the signals so that the EMGs and IMUs would have the same number of 1000 points for both sensors. However, only one window of 300 points was sent to the feature extraction stage to be evaluated at each time instant. Each EMG sensor had 8 channels, and to obtain the IMU signal, the 4 signals of the quaternions were used; thus, each EMG-IMU window information had a dimension of [300,12].

### 2.3. Feature Extraction

Feature extraction methods are used to extract relevant and non-redundant features from EMGs and IMUs. For this purpose, different domains can be used such as time, frequency, or time-frequency domains. In this work, five different features were extracted in the time domain over each step of the sliding window. The feature extraction functions used were root mean square (RMS), standard deviation (SD), energy (E), mean absolute value (MAV), and absolute envelope (AE), which are typically used to extract features of EMGs [1,14]. We used all these features in a feature vector since we obtained better results than when we used only one or a few of them. Since we had 5 feature extraction methods and an EMG-IMU window size of [300,12], a feature vector with a size of [60,1] was extracted from each of the EMG-IMU windows, which was made up of a feature vector with a size of [40,1] that corresponded to the EMGs and a vector with a size of [20,1] that corresponded to the quaternions obtained from the IMU.

### 2.4. Classification of EMGs

The objective of this stage is to identify the category of a hand gesture using an EMG-IMU signal among a set of categories with which the proposed algorithm was previously trained. In this work, we used an RL algorithm called deep Q-network (DQN), which is made up of a neural network to represent the agent’s policy. In this section, we explain in detail the EMG-IMU signal sequential classification problem that can be modeled as a partially observable finite Markov decision process (POMDP).

#### 2.4.1. Q-Learning

We can define the sliding window classification on an EMG-IMU signal sample during the development of a hand gesture as a sequential decision-making problem. In this problem, the actions correspond to the labels of the hand gestures to be inferred, whereas the states are the feature vectors corresponding to the observations of each window of an EMG-IMU sample. In this context, we can learn to estimate the optimal action for each state. For this purpose, we maximized the expected sum of future rewards by performing that action in the given states and then following an optimal policy [26]. Thus, considering a given policy π, the value of the action *a* taken in the initial state *s* can be defined as

(1) $$Q_{\pi }\left(s,a\right)=E_{\pi }\left[R_{1}+\gamma R_{2}+\gamma^{2}R_{3}+\ldots +\gamma^{n-1}R_{n}|S_{0}=s,A_{0}=a\right]$$

where $R_{i}$ are the rewards or punishments that the agent receives at each state with i=1, 2,…,n, where *n* represents the number of states. The variable γ ϵ [0,1] is the discount factor that determines how much future rewards affect the agent’s learning process. Then, the optimal state-action value function can be expressed as $Q_{*}\left(s,a\right)=max_{\pi }Q_{\pi }\left(s,a\right)$. An optimal policy can be calculated from the optimal function $Q_{*}\left(s,a\right)$ by choosing the highest valued action at each state according to [27]. Typically, to estimate the optimal state-action values, we can use the Q-learning algorithm, which is an off-policy temporal difference RL method [26]. For any finite Markov decision process (MDP), the Q-learning algorithm can find an optimal policy by maximizing the expected return function that we presented in Equation (1) given an initial state and an initial action [27]. However, it is important to consider that we assume that only the observations $O_{t}$ are measured instead of the complete state information of the environment $s_{t}$. This is because there may be a discrepancy between the set of EMG-IMU window observations and the set of feature vectors [16]. For this reason, in this work, we considered the HGR problem using EMG-IMU as a partially observable Markov decision process (POMDP) [16].

The Q-Learning algorithm uses Q-values to iteratively improve the behavior of the learning agent. The Q-values are an estimation of the performance of a certain action $A_{t}$ at the observation $O_{t}$. There are different ways to represent the Q-values such as polynomial functions, tables, or neural networks [27]. In the proposed method, we used a continuous observation space represented by the extracted EMG-IMU features and a discrete action space represented by the predicted hand gestures. Therefore, the Q-learning algorithm should be combined with a function approximation approach to learningc a parameterized value function $Q(O_{t},A_{t};\theta_{t})$. A critic representation can be used to obtain high-performance results when using discrete action spaces and continuous observations [27]. For a given observation and action, a critic agent output returns the expected value of the cumulative long-term reward. The standard Q-learning algorithm updates the parameters $\theta_{t}$ after taking action $A_{t}$ in observation $O_{t}$, obtaining the reward $R_{t+1}$ in $O_{t+1}$, described as follows:

(2) $$\theta_{t+1}=\theta_{t}+\alpha \left(Y_{t}^{Q}-Q\left(O_{t},A_{t};\theta_{t}\right)\right)\cdot \nabla_{\theta_{t}}Q\left(O_{t},A_{t};\theta_{t}\right)$$

Here, $\theta_{t+1}$ and $\theta_{t}$ are the updated and the previous parameters, respectively, and α is the learning rate. Finally, the target function $Y_{t}^{Q}$ is defined as

(3) $$Y_{t}^{Q}\equiv R_{t+1}+\gamma \cdot \underset{a}{max}\left[Q\left(O_{t+1},a;\theta_{t}\right)\right]$$

where the term $\underset{a}{max}\left[Q\left(O_{t+1},a\right)\right]$ is the estimated optimal future Q value. The term γ is the discount factor, and a reward $R_{t+1}$ is received by the agent when moving from the observation $O_{t}$ by taking the action $A_{t}$ to the next observation $O_{t+1}$.

#### 2.4.2. Deep Q-Networks (DQN)

In this work, we use a deep Q-network (DQN) agent representation, which is composed of an artificial neural network (ANN) as a function approximation method to learn a parameterized value function. Thus, for a given observation $O_{t}$, a DQN returns a vector of action values $Q(O_{t},\cdot \;;\theta )$, where θ are the parameters of the neural network [24,26,27]. The number of inputs of the network is the same as the dimension of the feature vector that represents an observation composed of the extracted EMG-IMU features [60,1], and the number of neurons at the output layer is the same as the number of possible actions that the agent can perform. According to [26,28], there are two key characteristics to consider in the DQN algorithm that are not considered in the standard Q-learning algorithm. The first is the use of a target network $Y_{t}^{DQN}$ that is used in Equation (4), which has parameters $\theta^{-}$ that are updated periodically every τ steps from the online network in Equation (2), with the parameters $\theta_{t}$. The rest of the time, the parameters $\theta^{-}$ remain fixed until the next update after τ steps. This helps to remove correlations with the target [26,28].

(4) $$Y_{t}^{DQN}\equiv R_{t+1}+\gamma \cdot \underset{a}{max}\left[Q\left(O_{t+1},a,\theta_{t}^{-}\right)\right]$$

The second important consideration is the use of experience replay, which randomly samples the data to remove correlations in the sequences of observations, which accelerates the training of the agent. For this purpose, the tuple $E_{t}=\left(O_{t},A_{t},R_{t},S_{t+1}\right)$ that represents the agent’s experience at time *t* is saved in a pool of stored data sample transitions $D=\{E_{1},E_{2},\cdots ,E_{T}\}$. During learning, the parameters of the ANN are updated using Equations (2) and (4), with the mini-batches of experience drawn uniformly at random from D [28,29]. The use of the target network with parameters $\theta^{-}$ and the experience replay approach help to significantly improve the performance of the DQN algorithm compared to the standard Q-learning algorithm [26,28]. The pseudo-code for the DQN algorithm is presented in Algorithm 1.
**Algorithm 1** DQN with Experience ReplayInitialize action-value function Q with random weightsInitialize replay memory D to capacity *N***for** episode = 1, M **do**    Initialize agent in observation $O_{t}$    **for** t = 1, T **do**        With probability ϵ select a random action $A_{t}$        otherwise, select $\underset{a}{max}\left[Q\left(O_{t+1},A,\theta_{t}^{-}\right)\right]$        store transition $E_{t}=\left(O_{t},A_{t},R_{t},S_{t+1}\right)$ in D        Sample random mini-batch of transitions $\left(O_{t},A_{t},R_{t},S_{t+1}\right)$ in D         $Y_{t}^{DQN}=\left\{\begin{array}{cc} R_{t+1} & for\;terminal\;O_{t}\\ R_{t+1}+\gamma \cdot \underset{a}{max}\left[Q\left(O_{t+1},a,\theta_{t}^{-}\right)\right] & for\;non-terminal\;O_{t} \end{array}\right.$        Perform gradient descent to update $\theta_{t+1}=\theta_{t}+\alpha \left(Y_{t}^{Q}-Q\left(O_{t},A_{t};\theta_{t}\right)\right)\cdot \nabla_{\theta_{t}}Q\left(O_{t},A_{t};\theta_{t}\right)$     **end for****end for**

#### 2.4.3. DQN for EMG-IMU Classification

**Figure 2:**
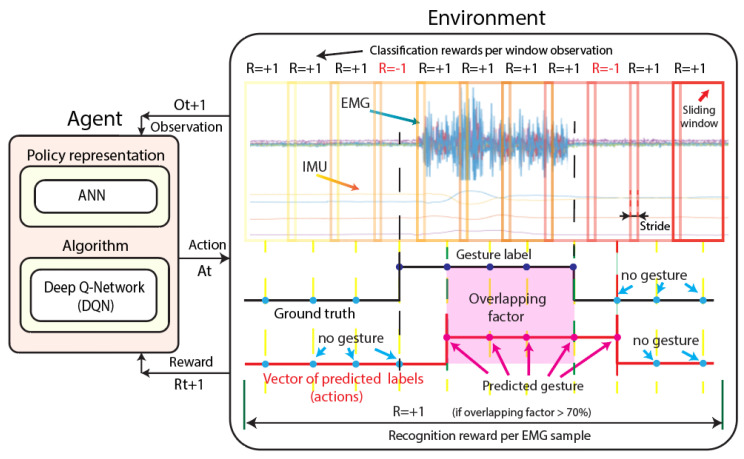
Scheme of the interaction between the DQN agent representation and the proposed environment for the EMG-IMU classification.

The proposed method modeled as a partially observable Markov decision process (POMDP) that we use in this work uses DQN the algorithm to learn an optimal policy, which allows an agent to learn to classify and recognize hand gestures from EMG-IMU signals. A figure that represents the interaction between the DQN agent representation and the proposed environment for the EMG-IMU classification is illustrated in Figure 2. We briefly explain each part of Figure 2 below.

Agent: The agent is made up of the DQN algorithm and an artificial neural network ANN as the policy representation. During training, the agent learns a policy that maximizes the total sum of rewards using the DQN algorithm. The inputs of the neural network are the features extracted from each window of the EMG-IMU signals (observations), and as its output, the network returns the values of the predicted gestures (actions). In this way, the agent learns to classify window observations from EMG-IMU signals. Each EMG-IMU signal sample is considered an independent episode, and each sliding window step is considered an observation during that episode.

Observation: The observation $O_{t}$ for a given unknown state $S_{t}$ is defined as the feature vector obtained from each EMG-IMU signal window. This vector is composed of RMS, SD, E, MAV, and AE information. The end of an episode occurs when the agent reaches the last sliding window observation of an EMG-IMU sample.

Action: An action $A_{t}$ is defined as the category of the gesture that the agent predicts to go from the current observation $O_{t}$ to the observation $O_{t+1}$, after which it receives a reward $R_{t+1}$. The categories of gestures used for this work are: wave in, wave out, fist, open, pinch, and relax (static gestures), and up, down, left, right, forward, and backward (dynamic gestures).

Environment: The environment is the defined environment within which the agent performs an action to move from one observation to the next, which returns a reward. In this case, we define the environment from the sliding window information—feature vectors and labels—extracted from each EMG-IMU signal and the ground truth (vector of known labels) of the EMG-IMU signal.

Reward: The agent receives a positive or negative reward depending on whether during its interaction with the environment it was able to correctly predict a gesture for a given observation. We define two different rewards, one for ranking and one for recognition. An illustration of the rewards that the agent obtains is presented in Figure 2. The agent can receive a positive reward $R_{t}=+1$ or a negative reward $R_{t}=-1$ depending on whether or not it correctly predicts the label of a window gesture. Once an episode ends, the vector of the known labels—ground-truth—is compared with the vector of the predicted labels, and if the overlapping factor between these vectors is greater than 70%, then recognition is considered successful and the agent receives a reward $R_{t}=+1$. If the recognition fails, the agent is penalized with $R_{t}=-1$.

### 2.5. Post-Processing

Once an EMG-IMU sample is processed and the vector of the predicted labels is obtained, we use post-processing to remove false labels and improve the accuracy of the proposed HGR system. There are several ways to perform post-processing such as using filters, majority voting, and heuristics, among others [1,16]. In this work, based on experimentation, we obtained the best results by calculating the mode on the vector of the predicted labels that are different from the relax labels. Then all the labels in those vectors that are different from the mode are replaced with it. The post-processing step is key to improving the classification and especially the recognition results since a single erroneous label in an EMG-IMU window can cause the recognition prediction to fail.

## 3. Results

In this section, we present the validation and testing results for the proposed HGR user-specific method for both the Myo armband and G-force sensors with regard to static and dynamic gestures. First, to find the best possible hyperparameters, we perform a validation procedure, and the best model results found during the validation are presented. Then, we present the final testing results with the previously found best hyperparameters. The validation and testing results for the Myo armband and G-force sensors are analyzed to compare their performance, considering separately static and dynamic gestures. Finally, we briefly compare the proposed method using the EMG-IMU signals with a similar method that uses only EMG.

### 3.1. Validation Results

For the validation results, we trained and tested different user-specific models based on an agent that uses neural networks as policy representations with the DQN algorithm that we presented previously in Section 2.4. For each model, we evaluated different hyperparameters such as the learning rate and mini-batch size to evaluate the classification and recognition results. Appendix A contains a summary of several of the tests performed to find the best hyperparameters. The best hyperparameter values found for the proposed method are summarized in Table 2.

**Figure 3:**
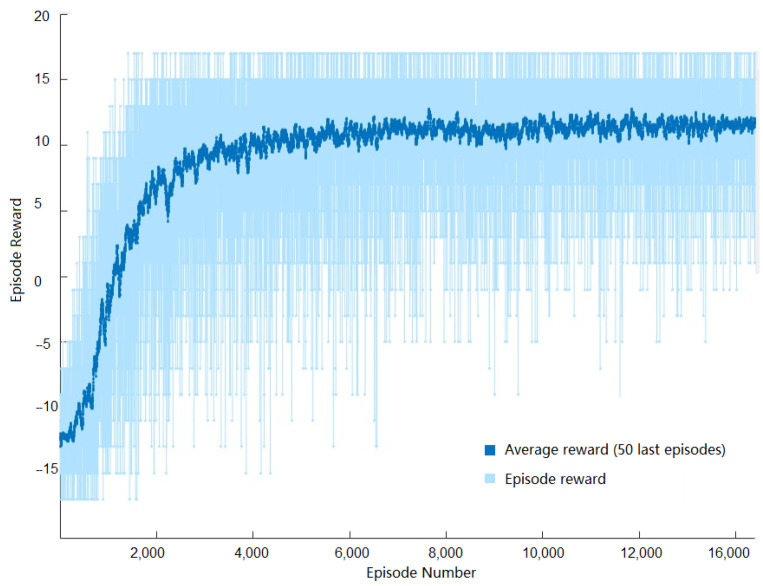
Sample of episode rewards versus episode numbers during the training of one user.

A training sample illustration of the average reward versus the number of episodes is illustrated in Figure 3. As can be observed, the curve in the figure shows satisfactory growth and convergence to the maximum average reward as the number of episodes increased. It is worth mentioning that this figure varied slightly depending on the data of each user. However, for all users, the same trend of convergence to the maximum average reward value was observed.

**Figure 4:**
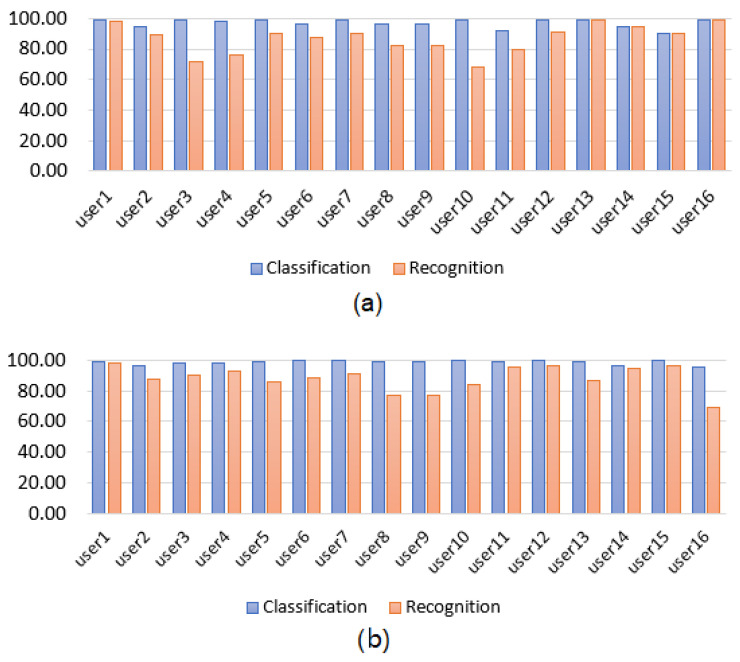
User-specific HGR model classification and recognition accuracy results for the Myo armband sensor using DQN. (**a**) Static gestures. (**b**) Dynamic gestures.

**Figure 5:**
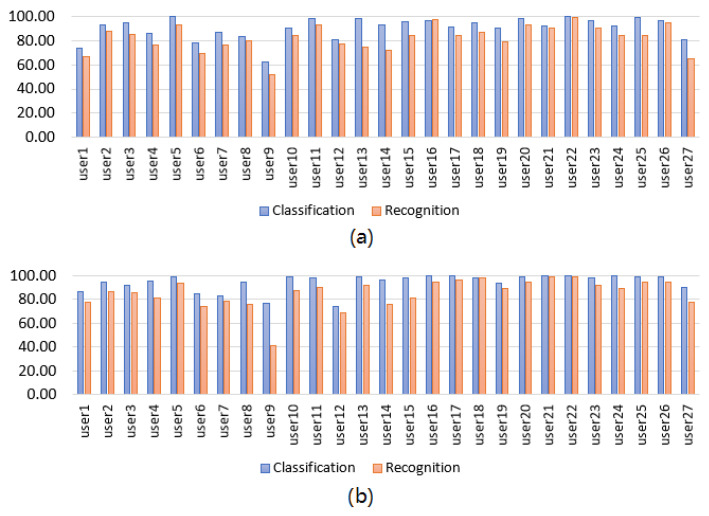
User-specific HGR model classification and recognition accuracy results for the G-force sensor using DQN. (**a**) Static gestures. (**b**) Dynamic gestures.

We present the classification and recognition results per user for the Myo armband sensor for static and dynamic gestures in Figure 4. Likewise, we present the classification and recognition results per user for the G-force sensor for static and dynamic gestures in Figure 5. Moreover, we present a summary of the best classification and recognition results of the user-specific HGR models obtained during validation in Table 3. It can be observed that for the validation results, the DQN-based model with the Myo armband sensor achieved slightly better results than the same model with the G-force sensor. There was a 6.5% classification accuracy difference between the Myo armband and G-force sensors for static gestures and a 4.3% difference for dynamic gestures. Moreover, the standard deviation was also lower for the Myo armband sensor, which was only 2.78% compared to a value of 9.04% for the G-force sensor. On the other hand, for dynamic gestures, the Myo obtained slightly better results. For example, for the Myo armband sensor, we obtained a 4.3% higher efficiency in the classification when using dynamic gestures with a standard deviation of only 1.37% compared to a value of 7.20% for the G-force sensor. The same analysis applied to the recognition accuracy metrics, demonstrating that the Myo armband sensor obtained slightly better results using this metric.

### 3.2. Testing Results

To present the testing results, we performed experiments on the test set based on the best hyperparameters previously found during the validation procedure presented in Section 3.1. This procedure helped us to evaluate our models with different data and analyze overfitting. We summarized the test results for 306 users with the best-found hyperparameters in Table 4. The classification results were similar for the two sensors, with the Myo-armband sensor obtaining slightly better results, with differences of 4.26% for static gestures and 1.82% for dynamic gestures compared to the G-force sensor. On the other hand, the recognition accuracy was similar for both sensors for the testing results compared with the validation results, with the exception of the G-force sensor, in which the recognition values were 56.45%±8.12% and 70.57%±11.99% for static and dynamic gestures, respectively. Overall, the testing classification results were similar to the validation results, demonstrating that the proposed models are robust to the effect of overfitting in terms of the classification of the proposed dataset distribution. Only for static gestures of the G-force sensor were the recognition results slightly lower. This is explained by the different distribution of the data and the variability of the users, as well as the fact that the hyperparameters were calibrated only for the validation dataset and not for the testing dataset.

**Figure 6:**
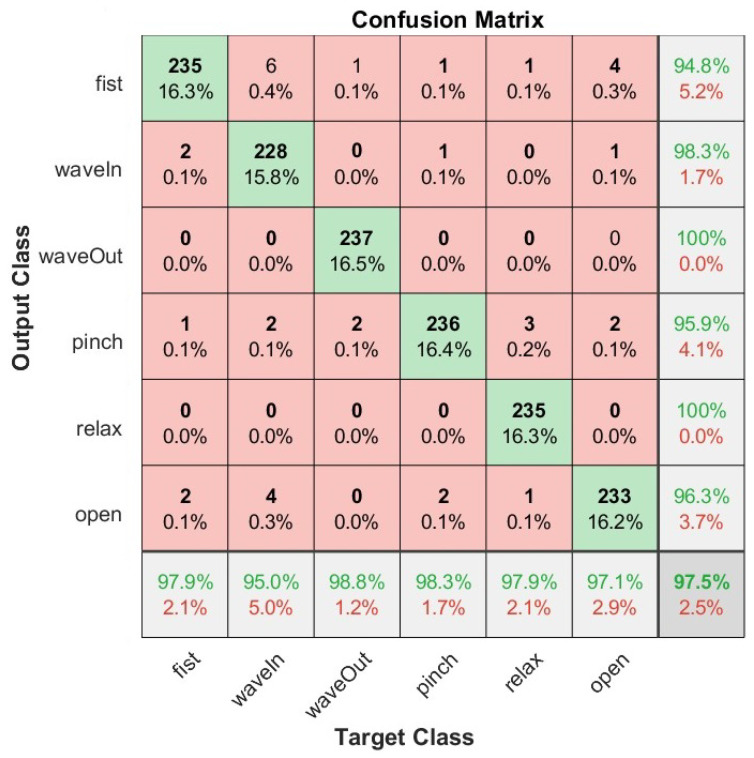
User-specific HGR model confusion matrix for 16 users from the test set with the best hyperparameter configuration for the Myo armband sensor for static gestures.

**Figure 7:**
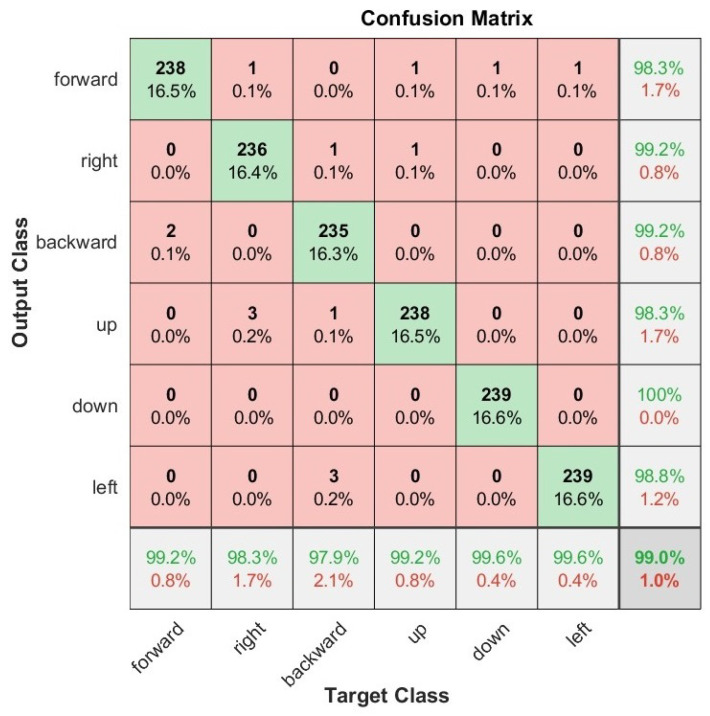
User-specific HGR model confusion matrix for 16 users from the test set with the best hyperparameter configuration for the G-force sensor for dynamic gestures.

**Figure 8:**
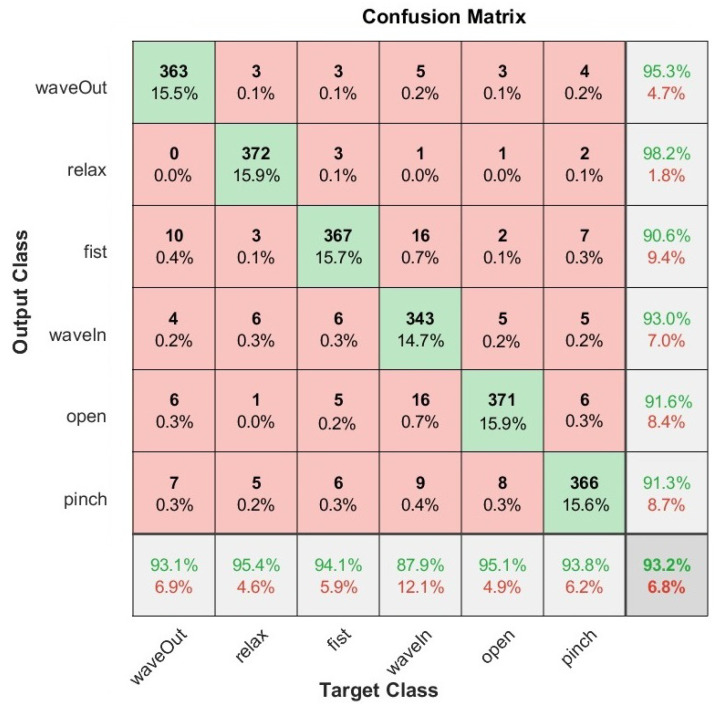
User-specific HGR model confusion matrix for 26 users from the test set with the best hyperparameter configuration for the Myo armband sensor for static gestures.

**Figure 9:**
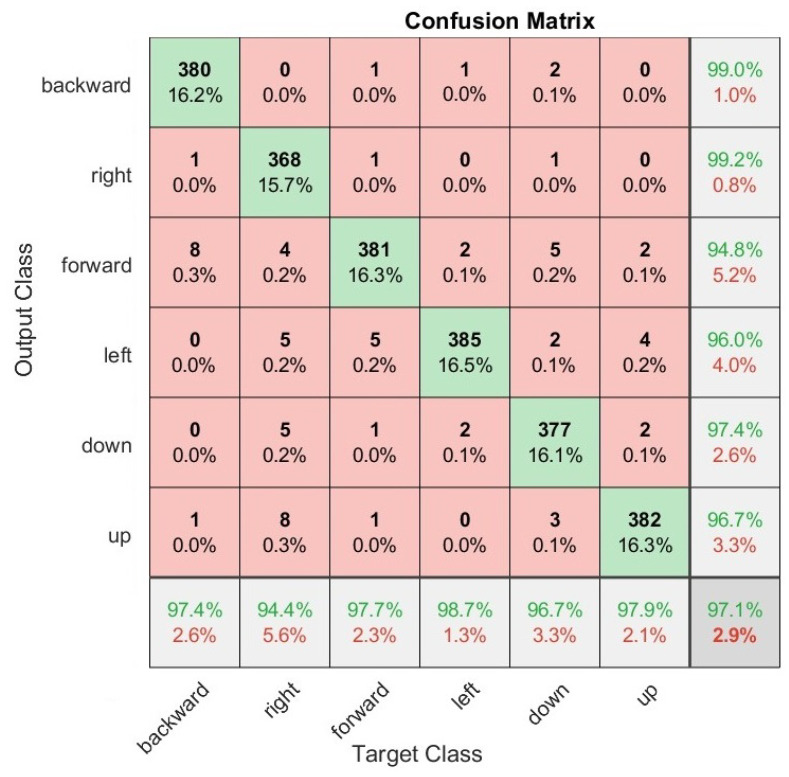
User-specific HGR model confusion matrix for 26 users from the test set with the best hyperparameter configuration for the G-force sensor for dynamic gestures.

We also present the confusion matrices that represent the classification results on the test set of the Myo armband sensor for static gestures in Figure 6 and dynamic gestures in Figure 7, as well as for the G-force sensor for static gestures in Figure 8 and dynamic gestures in Figure 9. In these figures, the results for each hand gesture can be observed in detail, which include both static and dynamic gestures for both sensors. It is worth mentioning that the processing time of each window observation was, on average, 33 ms.

### 3.3. Comparison with Other Methods

We implemented two additional tests for our proposed dataset and method, but the classification stage was based on supervised learning methods such as k-nearest neighbor (KNN) and a convolutional neural network (CNN). We also compared the results found in the present work, which uses EMG and IMU signals, with methods previously developed using the same sensor, with a similar dataset distribution with similar method stages that work with supervised and reinforcement learning [16,25]. These comparisons were useful for evaluating the effect of using EMG-IMU signals with respect to using EMG signals only, as well as comparing supervised and reinforcement learning methods for the proposed dataset. The selection criteria for the selected articles were based first on the type of sensor and its location on the user’s arm, which needs to be consistent with what we proposed in this work. Another important point that we considered is that we found that in the works based on EMGs only, the HGR models were trained to recognize static gestures only. To successfully recognize dynamic gestures, it was necessary to use IMU signals or a combination of IMU and EMG signals. This is because dynamic gestures are highly dependent on the user’s arm movements, which can be analyzed using information obtained from the IMU. We searched for approaches using similar methods that contained pre-processing, feature extraction, classification, and post-processing to fairly and objectively assess the effect of using EMG with IMU signals instead of just using EMG signals to develop HGR systems. The results using EMG and IMU signals that we obtained in this work for static gestures using the Myo armband sensor can be seen in Table 5, where we obtained 97.5%±1.13% and 88.15%±2.84% for the classification and recognition, respectively. On the other hand, another approach that used only EMG signals and Q-learning obtained 90.47%±14.24% and 87.51%±14.1% for the classification and recognition, respectively [16]. The approach that used EMG and IMU signals with supervised learning based on KNN obtained 80.04% and 66.12% for the classification and recognition, respectively, whereas the approach based on a CNN classifier obtained 84.49%±7.10% for the classification and 70.02%±8.21% for the recognition. Finally, another approach that used only EMG signals and a supervised learning approach based on a support vector machine obtained 95% and 81.6% for the classification and recognition, respectively [25]. As can be observed, using EMG and IMU signals helped to improve the classification and recognition results for static gestures when considering models based on reinforcement and supervised learning. Moreover, it can be observed that our model based on reinforcement learning with EMG and IMU signals presented the best results for this application.

## 4. Discussion

- According to the test results, the best classification accuracies were obtained for static gestures using the Myo armband sensor and were 97.50%±1.13% and 88.15%±2.84% for the classification and recognition, respectively. On the other hand, for dynamic gestures using the Myo armband sensor, the accuracies were 98.95%±0.62% and 90.47%±4.57% for the classification and recognition, respectively. The accuracies of the test results for static gestures using the G-force sensor were 93.24%±3.43% and 56.45%±8.12% for the classification and recognition, respectively. On the other hand, for dynamic gestures using the G-force sensor, the accuracies were 97.13%±2.04% and 70.57%±11.99% for the classification and recognition, respectively. This indicates that the method based on a DQN for the Myo armband sensor obtained slightly better results than the method based on a DQN for the G-force sensor.
- We compared the proposed method that used EMG and IMU signals with respect to other similar works where the same sensor was used with only EMG signals for static gestures. We obtained accuracies of 97.5%±1.13% and 88.15%±2.84% for the classification and recognition, respectively, using both EMG and IMU signals versus accuracies of 90.47%±14.24% and 87.51%±14.1% for the classification and recognition, respectively, using only EMG signals. This indicates the benefits of using EMG-IMU signals over using EMGs alone. This represents a 7% and 1% improvement in the classification and recognition, as well as a substantial reduction of more than 10% in the standard deviation of these metrics when using EMG-IMU signals instead of EMG signals alone. This also indicates the benefits of using EMG-IMU signals over using EMGs alone. Moreover, it can be seen that we are the first study to use RL with EMG-IMU signals to obtain better results compared to using only EMG signals with RL. Our results also outperformed those obtained with methods that use EMG or EMG-IMU with supervised learning.
- In general, the difference between the results of the validation and testing with regard to the classification and recognition was less than 5%. This difference is small so it can be said that the proposed method is robust and does not suffer from the effects of overfitting for the proposed dataset distribution.
- The processing time of each window observation was, on average, 33 ms for both sensors. Since this is less than 300 ms, we can consider that both models work in real time for the proposed application.
- Although the proposed results are encouraging, it is important to mention that in future works we will focus on the convenience and comfort that users experience when using static or dynamic gestures. User preference data can impact the development of HGR architectures so we will study this in depth in future work.

## 5. Conclusions

In this work, we proposed an HGR system based on the DQN algorithm for the classification of 11 different hand gestures including static and dynamic gestures. We tested and compared the results of two different sensors, the Myo armband and G-force sensors, from which we used the EMG and IMU signals to obtain the feature vectors. The proposed models were validated on 43 users and tested on 42 different users. The best classification accuracy was obtained for the Myo armband sensor, reaching up to 97.50%±1.13% and 88.15%±2.84% for the classification and recognition, respectively, with regard to static gestures, and 98.95%±0.62% and 90.47%±4.57% for the classification and recognition, respectively, with regard to dynamic gestures. The results obtained in this work showed that the DQN was able to learn a policy from online experience to classify and recognize gestures based on EMG and IMU signals, significantly improving the results obtained by similar methods using only EMG. It was also observed that the use of the Myo armband sensor compared to the G-force sensor obtained better accuracy for this application and data distribution. Future work includes testing other feature extraction methods and reinforcement learning algorithms to evaluate the proposed dataset.

## Figures and Tables

**Table 1:** Dataset distribution to evaluate user-specific models [25].

	User-Specific Model (One Model for Each of the 85 Users)			
	Number of Models	Training	Validation	Test
Training set	43 models trained (to find the best hyperparameters)	180 samples per user	180 samples per user	-
Testing set	42 models trained (to use the best of the found hyperparameters)	180 samples per user	-	180 samples per user

**Table 2:** Best hyperparameters found during validation procedure.

Hyperparameter Name	Hyperparameter Values
Activation function between layers	Relu
Target Smooth Factor	5 × 10${}^{-3}$
Experience buffer length	1 × 10${}^{6}$
Learn rate (α)	0.3 × 10${}^{-3}$
Epsilon initial value	1
Epsilon greedy epsilon decay	1 × 10${}^{-4}$
Discount factor	0.99
Training set replay per user	15 times
Sliding window size	300 points
Stride size	40 points
Mini-batch size	64
Optimizer	Adam
Gradient decay factor	0.9
L2 regularization factor	0.0001
Number of neurons for layer	60, 50, 50, 7 for the input layer, hidden layer 1, hidden layer 2, and output layer, respectively

**Table 3:** User-specific validation: best results for Myo armband and G-force sensors.

Sensor	Classification Accuracy	Recognition Accuracy
Myo armband (Static gestures)	96.9%±2.78%	87.0%±9.36%
Myo armband (Dynamic gestures)	98.6%±1.37%	88.2%±8.28%
G-force (Static gestures)	90.4%±9.04%	82.2%±10.98%
G-force (Dynamic gestures)	94.3%±7.20%	85.5%±12.3%

**Table 4:** User-specific testing results for Myo armband and G-force sensors.

Sensor	Classification Accuracy	Recognition Accuracy
Myo armband (Static gestures)	97.50%±1.13%	88.15%±2.84%
Myo armband (Dynamic gestures)	98.95%±0.62%	90.47%±4.57%
G-force (Static gestures)	93.24%±3.43%	56.45%±8.12%
G-force (Dynamic gestures)	97.13%±2.04%	70.57%±11.99%

**Table 5:** Comparison of classification and recognition accuracy results on the test set of the proposed model compared with other methods.

Learning Method	Type of Signal	Classification	Recognition
Reinforcement learning (this work)	EMG + IMU	** 97.5%±1.13% **	** 88.15%±2.84% **
Reinforcement learning [16]	EMG	90.47%±14.24%	87.51%±14.1%
Supervised learning—KNN classifier	EMG + IMU	80.04%±13.66%	66.12%±18.30%
Supervised learning—CNN classifier	EMG + IMU	84.49%±7.10%	70.02%±8.21%
Supervised learning [25]	EMG	95%	81.6%

## Data Availability

The dataset is available at https://laboratorio-ia.epn.edu.ec/en/resources/dataset/emg-imu-epn-100 accessed on 18 November 2022.

## Abbreviations

The following abbreviations are used in this manuscript:

HGR	hand gesture recognition systems
EMG	electromyography
EMGs	electromyography signals
IMU	inertial measurement unit
IMUs	inertial measurement unit signals
ML	machine learning
RL	reinforcement learning
CNN	convolutional neural network
ANN	artificial neural network
DQN	deep Q-network

## Appendix A

A summary of the validation results from changing the learning rate (alpha) parameter is presented in Table A1.

**Table A1:** User-specific validation results for Myo armband and G-force sensors.

Alpha	Classification Accuracy	Recognition Accuracy
0.07	39.2%±16.52%	25.0%±16.86%
0.05	38.9%±17.5%	22.0%±17.86%
0.03	45.9%±15.79%	37.0%±15.55%
0.01	51.9%±16.45%	47.0%±14.36%
0.007	54.2%±15.58%	48.6%±16.57%
0.005	70.5%±9.58%	57.2%±10.45%
0.003	71.4%±10.25%	58.2%±13.33%
0.001	77.3%±6.78%	73.4%±11.56%
0.0007	87.3%±4.11%	83.2%±12.22%
0.0005	89.2%±3.58%	75.2%±10.12%
**0.0003**	** 96.9%±2.78% **	** 87.0%±9.36% **
0.0001	93.2%±4.51%	83.5%±9.78%
0.00007	94.3%±3.58%	82.1%±8.35%
0.00005	88.3%±4.58%	80.1%±8.89%
0.00003	83.5%±6.52%	77.0%±13.48%
0.00001	85.3%±5.86%	81.0%±12.89%
